# Prognosis Comparison Between Nipple-Sparing Mastectomy and Total Mastectomy in Breast Cancer: A Case-Control Study After Propensity Score Matching

**DOI:** 10.1245/s10434-021-11044-4

**Published:** 2021-11-20

**Authors:** Mengdie Fu, Qitong Chen, Liyun Zeng, Tao Hong, Qiongyan Zou, Yunchang Yuan, Wenjun Yi

**Affiliations:** 1 Department of General Surgery, The Second Xiangya Hospital, Central South University, Changsha, China; 2 Department of Thoracic Surgery, The Second Xiangya Hospital, Central South University, Changsha, China

## Abstract

**Background:**

Currently, the operation rate of nipple-sparing mastectomy (NSM) is increasing. However, the long-term prognosis of NSM is not well documented. We utilized the Surveillance, Epidemiology, and End Results (SEER) database to analyze the long-term prognosis of NSM compared with total mastectomy (TM).

**Methods:**

Population-level data of female breast cancer patients treated with NSM and TM were extracted from 1998 to 2016 from the SEER database. Propensity score matching (PSM) was performed to reduce the influence of selection bias and confounding variables in comparisons. Kaplan-Meier analysis, log-rank test, and Cox proportional hazard regression were performed.

**Results:**

A total of 5765 patients underwent NSM, which increased from 266 in 2004–2009 to 5370 in 2010–2016. A total of 134,528 patients underwent TM, and the number of patients undergoing TM continued to decline. The overall survival (OS) and breast cancer-specific survival (BCSS) were similar between the NSM group and the TM group (*P* = 0.058 and 0.87, respectively). For OS, subgroup analysis showed that patients with age ≥ 46, White race, median household income ≥ $70,000, hormone receptor-positive, and HER2 negative had a better prognosis for treatment with NSM. There was no significant difference in BCSS between the NSM group and the TM group.

**Conclusions:**

In recent years, the clinical application of NSM has been increasing. NSM is a proper procedure for breast cancer patients to achieve long-term survival.

**Supplementary Information:**

The online version contains supplementary material available at 10.1245/s10434-021-11044-4.

Breast cancer is the most common cancer in women and the main cause of cancer death.^1,2^ From the concept of breast conservation proposed by Bernard Fisher in the 1980s to the advent of sentinel lymph node (SLN) assessment and skin-sparing mastectomy in the 1990s, surgery has become more conservative. The development of nipple retention techniques in the late 1990s and early 2000s was justified, as studies demonstrated a lower risk of involvement of the nipple and areola complex (NAC) in selected cancer patients.^3–5^ In 1999, Lynn Hartman published a paper in the New England Journal of Medicine^6^ showing that prophylactic mastectomy, better known as reduced-risk nipple-sparing mastectomy (NSM), was beneficial in high-risk patients with a 90% reduction in the incidence of breast cancer. This first started the movement of the nipple retention method. In recent years, the demand for NSM has increased due to the enhanced aesthetic effects provided by natural NAC. NSM refers to the removal of all visible breast tissue and submission of subpapillary duct tissue for histological evaluation. That is, NSM excises the breast parenchyma to the same extent as conventional total mastectomy (TM). The 2020 National Comprehensive Cancer Network (NCCN) guidelines recommend that NSM is optional in oncology, except for the following contraindications: Paget’s disease, bloody nipple discharge associated with malignant tumors, inflammatory breast cancer and/or imaging findings suggesting that the nipple or subareolar tissue is involved in malignant lesions.^7^

The prognosis of NSM in breast cancer is being continuously explored and proven. Alessio Meter et al.^8^ studied complications and recurrence rates in 894 patients who underwent NSM between 2002 and 2017. The mean follow-up time was 41.2 months. The majority of patients treated with NSM did not have any early or late complications (83.2% and 65.8%), and the local recurrence rate in the NAC and skin was 4.9%. Victor Lago et al.^9^ reviewed 69 NSM patients diagnosed with ductal carcinoma in situ (DCIS) between 1984 and 2016, with a median follow-up time of 142.6 months. A low nipple recurrence rate (1.4%) and high survival rate (98.5%) were observed. No nipple necrosis was observed. Thus, they concluded that NSM is a realistic treatment option for patients with DCIS who are not suitable for breast-conserving therapy. Zoranrado Vanovic et al.^10^ conducted a retrospective study of 435 patients who underwent 441 NSM surgeries from 2004 to 2012. Local recurrence occurred in 32 patients (7.3%), distant metastasis was diagnosed in 68 patients (15.6%), and 53 patients (12.2%) died during follow-up. Barbara L. Smith et al.^11^ evaluated the long-term outcomes of 311 patients with stage 0–3 breast cancer undergoing NSM from 2007 to 2012. At a median follow-up of 51 months, 17 patients had cancer recurrence. The estimated 3-year and 5-year disease-free survival rates were 95.7% and 92.3%, respectively. Local recurrence occurred in 11 patients (3.7%) and distant recurrence in 8 patients (2.7%); local and distant recurrence occurred simultaneously in 2 patients. It was concluded that the local and distant recurrence rates of breast cancer patients after NSM were low. Violette Mesdag et al.^12^ studied the prognosis and patient satisfaction of 63 breast cancer patients who underwent NSM and 89 breast cancer patients who underwent skin-sparing mastectomy (SSM). The median follow-up time was 42 months. In the NSM group, only one patient had local recurrence, but it did not involve the preserved nipple. The disease-free survival rate after 3 years in the NSM group was 97.6% [95% confidence interval (CI): 84.3–99.7] (*P* = 0.72). The patients were satisfied with NSM for the treatment of cancer (76.8%).

However, there is a lack of large-sample studies on the long-term outcomes (overall survival (OS) and breast cancer-specific survival (BCSS) of NSM. Therefore, our study aims to evaluate the long-term prognosis and survival benefits of NSM in female patients with M0 stage breast cancer based on data from the Surveillance, Epidemiology, and End Results (SEER) database.

## Materials and Methods

### Data Source

Population-level data were extracted from the National Cancer Institute’s SEER cancer database (http://www.seer.cancer.gov) via SEER*Stat software (https://seer.cancer.gov/seerstat/, version 8.3.8). The SEER database collects patient-level data of all cancer indexes from 18 cancer registries in the United States, accounting for 28% of the national population.^13^ Each person was diagnosed, and the SEER registry collected primary demographic data, tumor clinicopathological characteristics, treatment mode, and survival status (including the cause of death of the patient during follow-up). This study was deemed exempt from review by the Ethics Committee of the Second Xiangya Hospital of Central South University because of the use of deidentified records.

### Patient Selection

Female patients diagnosed with pathologically confirmed breast cancer who underwent NSM (SEER surgery code 30) or TM (codes 40–49,75) from 1998 to 2016 in the SEER program database were enrolled in the study. The exclusion criteria were as follows: (1) not primary tumor; (2) had incomplete follow-up data; and (3) presence of disease other than AJCC M0 stage disease (M1 or MX). All data collection and coding rules for data collection are specified by the code of the SEER program coding and staging manual.^14^ Ultimately, a total of 140,293 female patients with primary breast cancer without distant metastasis were screened.

### Statistical Analysis

This study was a retrospective observational study, so surgery assignment was not randomized. Many clinical prognostic factors such as age, marital status, race, median household income, year of diagnosis, grade, T stage, N stage, histology, estrogen receptor (ER) status, progesterone receptor (PR) status, human epidermal growth receptor 2 (HER2) status and molecular subtype were heterogeneous between the NSM patients and TM patients in the SEER database. We implemented propensity score matching (PSM)^15^ using the R package “MatchIt”^16^ version 4.1.0 with the following settings: 1:3 pairing, nearest-neighbor methods, and a caliper of 0.02 to balance the baseline characteristics of patients with NSM and TM treatment. (For more details, please refer to Supplementary File 1). After PSM, the following demographic and clinicopathological characteristics of breast cancer patients were well balanced and were included in further analysis: age, marital status, race, median household income, year of diagnosis, laterality, tumor grade, T stage, N stage, tumor histology, ER status, progesterone receptor (PR) status, human epidermal growth receptor 2 (HER2) status, molecular subtype. The patients were divided into two main subgroups: NSM and TM.

Using the chi-square test, we compared the demographic and clinicopathological characteristics of NSM and TM patients. OS was defined as the time from diagnosis to death from any cause, and BCSS was defined as the time from the initial diagnosis to breast cancer-related death. OS and BCSS were the primary endpoints of this study. The Kaplan-Meier method was used to estimate the 5-year and 10-year OS and BCSS.^17^ Cox proportional hazard regression analysis was utilized to calculate the risk ratio and 95% CI, and the results are shown in the forest plot. Statistical analyses and data visualization were performed using R (https://www.r-project.org/, version 4.0.3). All statistical tests were two-sided, and the statistical significance level was set at *P* < 0.05.

## Results

### Demographic Characteristics

**Fig. 1.**
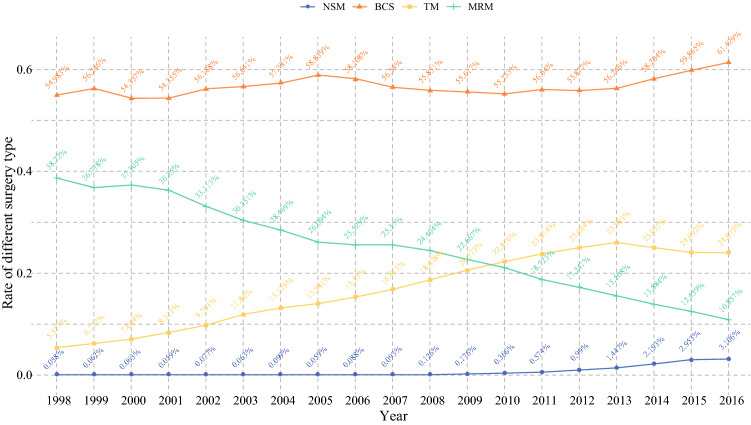
Changes in the rate of different surgical types for breast cancer from 1998 to 2016

Between 1998 and 2016, 140,293 female patients in the SEER database underwent mastectomy-coded NSM (5765, 4.1%) and TM (134,528, 95.9%). We visually assessed the changes in four basic surgical methods from 1998 to 2016. The proportion of NSM increased steadily, while that of TM began to decline after 2013 (Fig. 1). Most of the patients treated with NSM were 46–65 years old (3213, 35.3%) and married (3857, 66.9%). Most of the patients treated with NSM were White (4472, 77.6%), and a small number (506, 8.8%) were Black. The overall median household income of patients receiving treatment ranged from $50,000 to $70,000 (Table 1).

**Table 1:** Baseline characteristics of patients with breast cancer

Category	Before propensity score matching				After propensity score matching			
	No. of patients (%)	NSM (%)	TM (%)	*P* value	No. of patients (%)	NSM (%)	TM (%)	*P* value
*Age*								
≤45	28,732 (20.5)	2036 (35.3)	26,696 (19.8)	<0.001	8017 (34.8)	2034 (35.3)	5983 (34.6)	0.559
46–65	69,471 (49.5)	3213 (55.7)	66,258 (49.3)		12,992 (56.4)	3213 (55.8)	9779 (56.6)	
> 65	42,090 (30.0)	516 (9.0)	41,574 (30.9)		2043 (8.9)	516 (9.0)	1527 (8.8)	
*Marital status*								
Married	82,324 (58.7)	3857 (66.9)	78,467 (58.3)	<0.001	15,654 (67.9)	3855 (66.9)	11,799 (68.2)	0.208
Single	19,386 (13.8)	879 (15.2)	18,507 (13.8)		3392 (14.7)	879 (15.3)	2513 (14.5)	
DSW	32,827 (23.4)	828 (14.4)	31,999 (23.8)		3262 (14.2)	828 (14.4)	2434 (14.1)	
Unknown	5756 (4.1)	201 (3.5)	5555 (4.1)		744 (3.2)	201 (3.5)	543 (3.1)	
*Race*								
White	111,677 (79.6)	4472 (77.6)	107,205 (79.7)	<0.001	18,003 (78.1)	4471 (77.6)	13,532 (78.3)	0.162
Black	12,846 (9.2)	506 (8.8)	12,340 (9.2)		1974 (8.6)	506 (8.8)	1468 (8.5)	
Other	14,994 (10.7)	741 (12.9)	14,253 (10.6)		2934 (12.7)	740 (12.8)	2194 (12.7)	
Unknown	776 (0.6)	46 (0.8)	730 (0.5)		141 (0.6)	46 (0.8)	95 (0.5)	
*Median household income ($)*								
<50,000	31,537 (22.5)	905 (15.7)	30,632 (22.8)	<0.001	3636 (15.8)	905 (15.7)	2731 (15.8)	0.981
50,000–70,000	70,051 (49.9)	2896 (50.2)	67,155 (49.9)		11,587 (50.3)	2896 (50.3)	8691 (50.3)	
≥70,000	38,705 (27.6)	1964 (34.1)	36,741 (27.3)		7829 (34.0)	1962 (34.0)	5867 (33.9)	
*Year of diagnosis*								
1998–2003	17,503 (12.5)	129 (2.2)	17,374 (12.9)	<0.001	518 (2.2)	129 (2.2)	389 (2.2)	0.902
2004–2009	40,995 (29.2)	266 (4.6)	40,729 (30.3)		1089 (4.7)	266 (4.6)	823 (4.8)	
2010–2016	81,795 (58.3)	5370 (93.1)	76,425 (56.8)		21,445 (93.0)	5368 (93.1)	16,077 (93.0)	
*Laterality*								
Left	71,370 (50.9)	2892 (50.2)	68,478 (50.9)	0.513	11,592 (50.3)	2891 (50.2)	8701 (50.3)	0.925
Right	68,889 (49.1)	2872 (49.8)	66,017 (49.1)		11,457 (49.7)	2871 (49.8)	8586 (49.7)	
Both sides	34 (0.0)	1 (0.0)	33 (0.0)		3 (0.0)	1 (0.0)	2 (0.0)	
*Grade*								
I–II	85,802 (61.2)	3611 (62.6)	82,191 (61.1)	0.004	14,572 (63.2)	3610 (62.6)	10,962 (63.4)	0.248
III–V	46,618 (33.2)	1881 (32.6)	44,737 (33.3)		7475 (32.4)	1881 (32.6)	5594 (32.4)	
Unknown	7873 (5.6)	273 (4.7)	7600 (5.6)		1005 (4.4)	272 (4.7)	733 (4.2)	
*T stage*								
T0	56 (0.0)	2 (0.0)	54 (0.0)	<0.001	6 (0.0)	2 (0.0)	4 (0.0)	0.091
T1	76,535 (54.6)	3348 (58.1)	73,187 (54.4)		13,492 (58.5)	3346 (58.1)	10,146 (58.7)	
T2	47,027 (33.5)	1901 (33.0)	45,126 (33.5)		7646 (33.2)	1901 (33.0)	5745 (33.2)	
T3	10,041 (7.2)	377 (6.5)	9664 (7.2)		1462 (6.3)	377 (6.5)	1085 (6.3)	
T4	3368 (2.4)	55 (1.0)	3313 (2.5)		191 (0.8)	55 (1.0)	136 (0.8)	
TX	3266 (2.3)	82 (1.4)	3184 (2.4)		255 (1.1)	82 (1.4)	173 (1.0)	
*N stage*								
N0	97,076 (69.2)	3948 (68.5)	93,128 (69.2)	<0.001	15,945 (69.2)	3947 (68.5)	11,998 (69.4)	0.091
N1	30,654 (21.8)	1390 (24.1)	29,264 (21.8)		5527 (24.0)	1390 (24.1)	4137 (23.9)	
N2	6187 (4.4)	249 (4.3)	5938 (4.4)		979 (4.2)	249 (4.3)	730 (4.2)	
N3	3110 (2.2)	97 (1.7)	3013 (2.2)		347 (1.5)	97 (1.7)	250 (1.4)	
NX	3266 (2.3)	81 (1.4)	3185 (2.4)		254 (1.1)	80 (1.4)	174 (1.0)	
*Histology*								
Ductal carcinoma	98,407 (70.1)	4274 (74.1)	94,133 (70.0)	<0.001	16,950 (73.5)	4274 (74.2)	12,676 (73.3)	0.429
Lobular carcinoma	26,176 (18.7)	959 (16.6)	25,217 (18.7)		3948 (17.1)	958 (16.6)	2990 (17.3)	
Other	15,710 (11.2)	532 (9.2)	15,178 (11.3)		2154 (9.3)	531 (9.2)	1623 (9.4)	
*ER*								
Positive	108,072 (77.0)	4684 (81.2)	103,388 (76.9)	<0.001	18,777 (81.5)	4683 (81.3)	14,094 (81.5)	0.753
Negative	25,219 (18.0)	969 (16.8)	24,250 (18.0)		3856 (16.7)	969 (16.8)	2887 (16.7)	
Unknown	7002 (5.0)	112 (1.9)	6890 (5.1)		419 (1.8)	111 (1.9)	308 (1.8)	
*PR*								
Positive	92,554 (66.0)	4155 (72.1)	88,399 (65.7)	<0.001	16,674 (72.3)	4153 (72.1)	12,521 (72.4)	0.315
Negative	39,312 (28.0)	1479 (25.7)	37,833 (28.1)		5910 (25.6)	1479 (25.7)	4431 (25.6)	
Unknown	8427 (6.0)	131 (2.3)	8296 (6.2)		468 (2.0)	131 (2.3)	337 (1.9)	
*HER2*								
Positive	13,527 (9.6)	928 (16.1)	12,599 (9.4)	<0.001	3633 (15.8)	927 (16.1)	2706 (15.7)	0.397
Negative	63,293 (45.1)	4196 (72.8)	59,097 (43.9)		16,902 (73.3)	4195 (72.8)	12,707 (73.5)	
Unknown	4975 (3.5)	246 (4.3)	4729 (3.5)		910 (3.9)	246 (4.3)	664 (3.8)	
Unavailable	58,498 (41.7)	395 (6.9)	58,103 (43.2)		1607 (7.0)	395 (6.9)	1212 (7.0)	
*Molecular subtype*								
HR+/HER2-	54,513 (38.9)	3649 (63.3)	50,864 (37.8)	< 0.001	14,664 (63.6)	3648 (63.3)	11,016 (63.7)	0.861
HR+/HER2+	9433 (6.7)	666 (11.6)	8767 (6.5)		2587 (11.2)	665 (11.5)	1922 (11.1)	
HER2 enriched	4070 (2.9)	261 (4.5)	3809 (2.8)		1043 (4.5)	261 (4.5)	782 (4.5)	
TNBC	8696 (6.2)	545 (9.5)	8151 (6.1)		2223 (9.6)	545 (9.5)	1678 (9.7)	
Unknown	63,581 (45.3)	644 (11.2)	62,937 (46.8)		2535 (11.0)	644 (11.2)	1891 (10.9)	
Total	140,293	5765 (4.1)	134,528 (95.9)		23,052	5763 (25.0)	172,89 (75.0)	

*NSM* nipple-sparing mastectomy; *TM* total mastectomy; *DSW* divorced/separated/widowed; *ER* estrogen receptor; *PR* progesterone receptor; *HER2* human epidermal growth receptor 2; *HR* hormone receptor; *TNBC* triple-negative breast cancer

### Clinicopathological Characteristics

In patients undergoing NSM, grade I–II was reported in the majority of patients (3611, 62.6%). Overall, T1 (3348, 58.1%) and N0 (3948, 68.5%) were the most abundant stages. Surgical laterality was left in 50.2% (2892/5764) of patients. A total of 81.2% of patients were ER positive, 72.1% of patients were PR positive, and 16.1% of patients were HER2 positive. Among the available molecular subtype data, HR+/HER2- (3649, 63.3%) was the most common. The same trend was observed in patients undergoing TM (Table 1).

### Radiation and Chemotherapy

A total of 17.2% of patients underwent adjuvant radiation therapy. Among the patients who underwent NSM, 22.3% received adjuvant radiotherapy and 77.7% had a nonradiation/unknown status. Among the patients who underwent TM, 17.0% received adjuvant radiotherapy, and 83.0% had a nonradiation/unknown status. In contrast, more patients received chemotherapy. A total of 43.4% of the patients underwent adjuvant chemotherapy. Among the patients who underwent NSM or TM, 49.9% and 43.2%, respectively, received adjuvant radiotherapy (Table 1).

### Survival Analysis

**Fig. 2.**
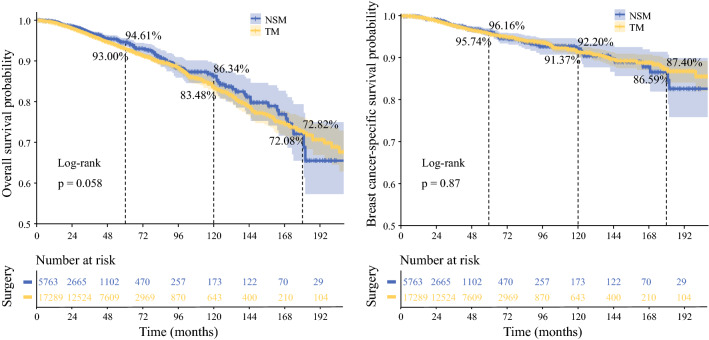
Kaplan-Meier curves of overall survival (OS) and breast cancer-specific survival (BCSS) for breast cancer patients who underwent NSM and TM

After PSM matching, 5763 patients receiving NSM and 17,289 patients receiving TM were included in the analysis (Table 1). It was noted that the 5-year (94.61% vs 93.00%) and 10-year (86.34% vs 83.48%) OS rates of the NSM group were higher than those of the TM group and that the 5-year (96.16% vs 95.74%) and 10-year (92.20% vs 91.37%) BCSS rates of the NSM group were higher than those of the TM group (Fig. 2). Kaplan-Meier survival curves and log-rank test indicated that the OS and BCSS were similar between the NSM group and the TM group (*P* = 0.058 and 0.87, respectively).

Survival analysis showed that age, marital status, race, median household income, tumor grade, N stage, ER status, PR status, molecular subtype, radiation, and chemotherapy were significant factors for the OS and BCSS of patients treated with NSM. At the same time, year of diagnosis and HER2 status were significant factors for OS, and histology was a significant factor for the BCSS (*P* < 0.05) (Supplementary Fig. 1 A–N). For OS, patients with age > 65, single marital status, Black race, low-median household income (< $50,000), diagnosed from 1998 to 2003, tumor grade III–IV, N3 stage, ER negative, PR negative, HER2 enriched, triple negative breast cancer (TNBC) subtype, radiotherapy and chemotherapy had worse prognosis. For BCSS, patients with Black race, tumor grade III–IV, N3, lobular carcinoma, ER negative, PR negative, TNBC, radiotherapy and chemotherapy had a worse prognosis.

### Subgroup Analysis

**Fig. 3.**
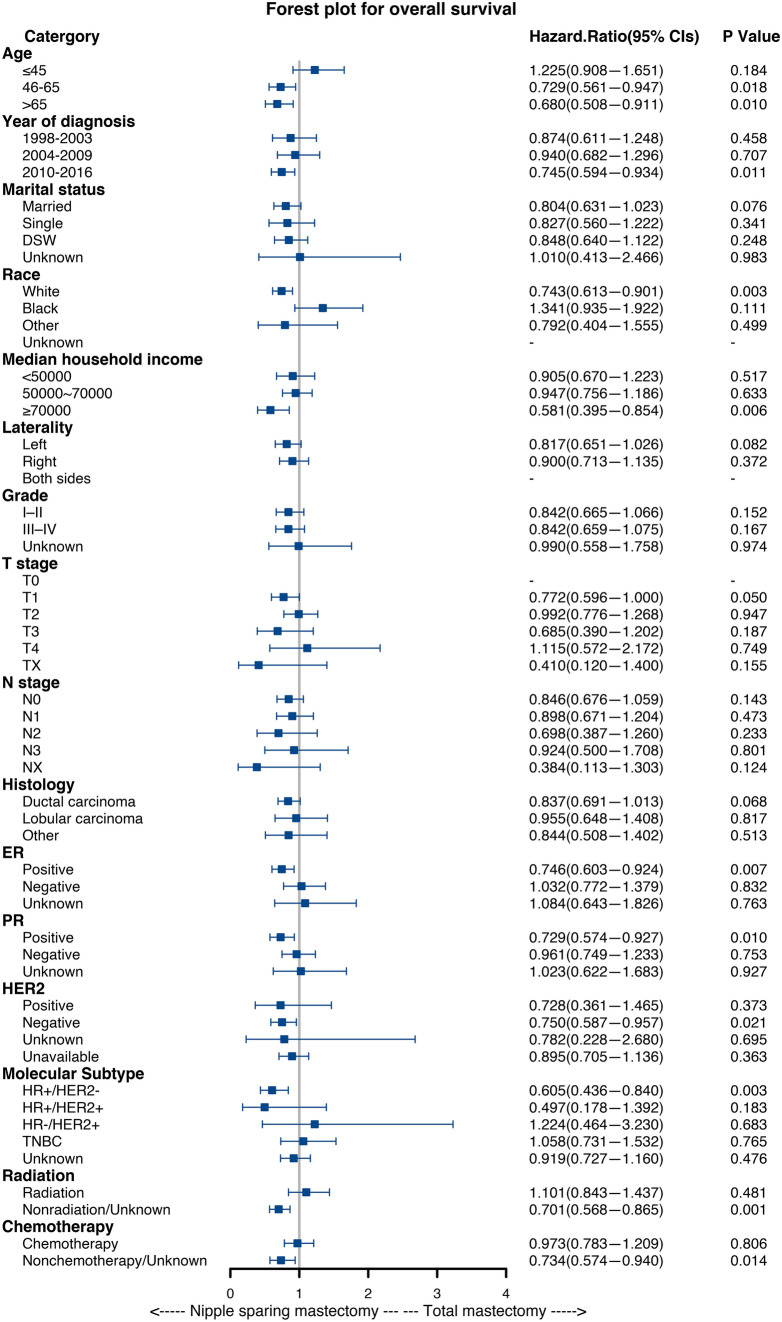
Forest plot for breast cancer patients in the subgroup analysis (NSM vs TM). Hazard ratio (HR) with 95% confidence interval (CI) for death in terms of the overall survival (OS) of patients with breast cancer who underwent NSM or TM. *P*-values of the Cox proportional hazards regression are reported

**Fig. 4.**
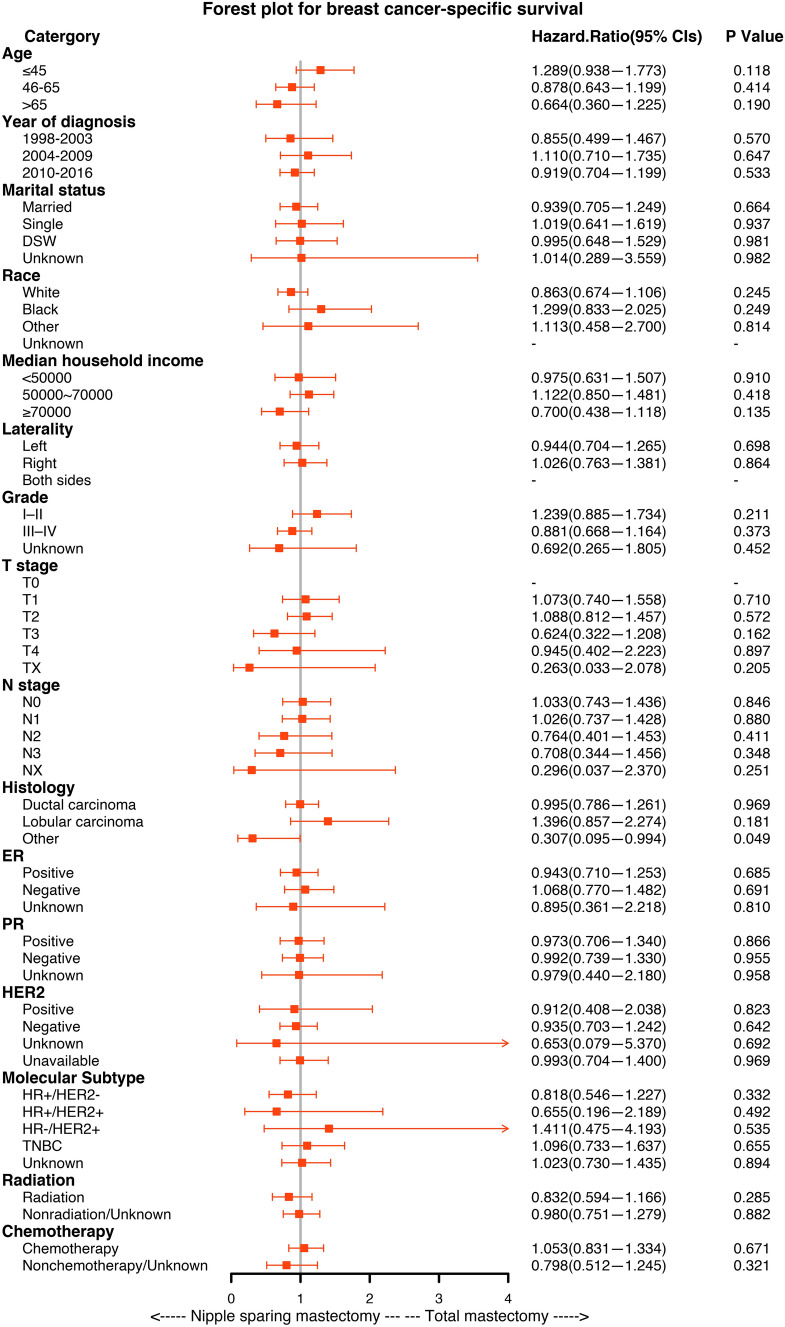
Forest plot for breast cancer patients in the subgroup analysis (NSM vs TM). Hazard ratio (HR) with 95% confidence interval (CI) for death in terms of the breast cancer-specific survival (BCSS) of patients with breast cancer who underwent NSM or TM. *P*-values of the Cox proportional hazards regression are reported

For OS, the forest plot showed that there was a significant difference when comparing the efficacy of NSM and TM. Some of the variables showed that NSM was beneficial for breast cancer patients compared with TM (Fig. 3), including age > 46, White race, median household income ≥ $70,000, ER positive,PR positive, HER2 negative, HR+/HER2- subtype, nonradiotherapy, and nonchemotherapy (*P* < 0.05). For BCSS, none of the subgroups showed significant differences (Fig. 4). This means that NSM was non-inferior to TM. These results may indicate that NSM has similar prognostic value compared with TM in breast cancer patients and shows greater advantages in some subgroups.

## Discussion

Our study analyzed the representative SEER database to describe the application of NSM and TM in the United States. The registries of the SEER program routinely collect demographic characteristics, clinicopathological characteristics, and survival status follow-up data. In this study, 140,293 female patients were screened, representing the largest reported group of NSM- and TM-treated patients in the United States. We used PSM to balance the baseline characteristics of the patients treated with NSM and TM.

In recent years, hesitation to provide NSM to cancer patients has stemmed from concern about the increased risk of local recurrence and the possibility that breast cancer will occur in breast epithelial tissue retained in NAC in the future. In one study with a median follow-up of > 5 years, the local recurrence rate was 2–11.7%, and the NAC recurrence rate was 1.3–3.7%.^18–20^ In the early years, there were 16 studies^20–35^ with a total of 1200 patients who underwent NSM. The OS rate ranged from 94.3 to 100%, and the NAC recurrence rate ranged from 0 to 3.6%.

Kaplan-Meier analysis and forest plots of subgroup analyses for OS and BCSS indicated that NSM is an important prognostic factor for breast cancer patients. It is worth noting that there were no significant differences between NSM and TM in the HER2-positive, HR+/HER2+ and HER2-enriched subgroups, but NSM improved the OS of breast cancer in the HER2-negative and HR+/HER2- subgroups. For radiotherapy and chemotherapy, which are common auxiliary clinical treatments, NSM improved OS in the nonchemotherapy and nonradiotherapy groups. This finding suggests that NSM may achieve a better prognosis for these special populations of breast cancer.

Our research also has limitations. First, this was a retrospective study with the possibility of selection bias, even though we utilized PSM statistical methods to diminish it and make our results more reliable. Second, in this study, nipple-sparing mastectomy surgery code 30 was utilized to identify all patients who had undergone NSM according to the SEER coding manual. However, it is important to note that the term ‘‘nipple-sparing mastectomy’’ was coded as a TM with the 'subcutaneous mastectomy' code in 1998–2010. Our study included patients from 1998 to 2016, and therefore it is possible that some patients receiving NSM were not appropriately coded as having undergone a subcutaneous mastectomy. Nevertheless, SEER surgery code 30 is the only code available for NSM with a clear definition on the SEER coding manual since 2011 and is the appropriate code for identifying these patients.^36^ Therefore, in Supplementary Fig. 2, we complemented Kaplan-Meier curves of prognosis comparison. It likewise indicated that NSM is a non-inferior procedure to TM both in 1998–2010 groups and 2011–2016 groups. Last, we included a small number of patients with bilateral NSM because SEER captured more data on nipple preservation during bilateral mastectomy. However, studies have shown the benefits of bilateral preventive NSM for patients with hereditary breast cancer,^37^ and it is a less invasive method for the prevention of primary breast cancer in high-risk women.^38^ Therefore, comparing the results of bilateral NSM and bilateral non-NSM has clinical value. Further high-quality and long-term follow-up trials should be conducted to verify our findings.

## Conclusions

In recent years, the clinical application of NSM has been increasing. NSM is a proper procedure for breast cancer patients to achieve long-term survival.

## Supplementary Information

Below is the link to the electronic supplementary material.

Supplementary file1 (DOCX 5274 KB)

Supplementary file2 (DOCX 125 KB)
