# Supplementary material for: Prognosis Comparison Between Nipple-Sparing Mastectomy and Total Mastectomy in Breast Cancer: A Case-Control Study After Propensity Score Matching
Source: Ann Surg Oncol. 2021 Nov 20;29(4):2221–30. doi: 10.1245/s10434-021-11044-4 (PMC8933300; doi:10.1245/s10434-021-11044-4)
Supplement: Supplementary file 1 — Supplementary file1 (DOCX 5274 KB) [file 10434_2021_11044_MOESM1_ESM.docx]

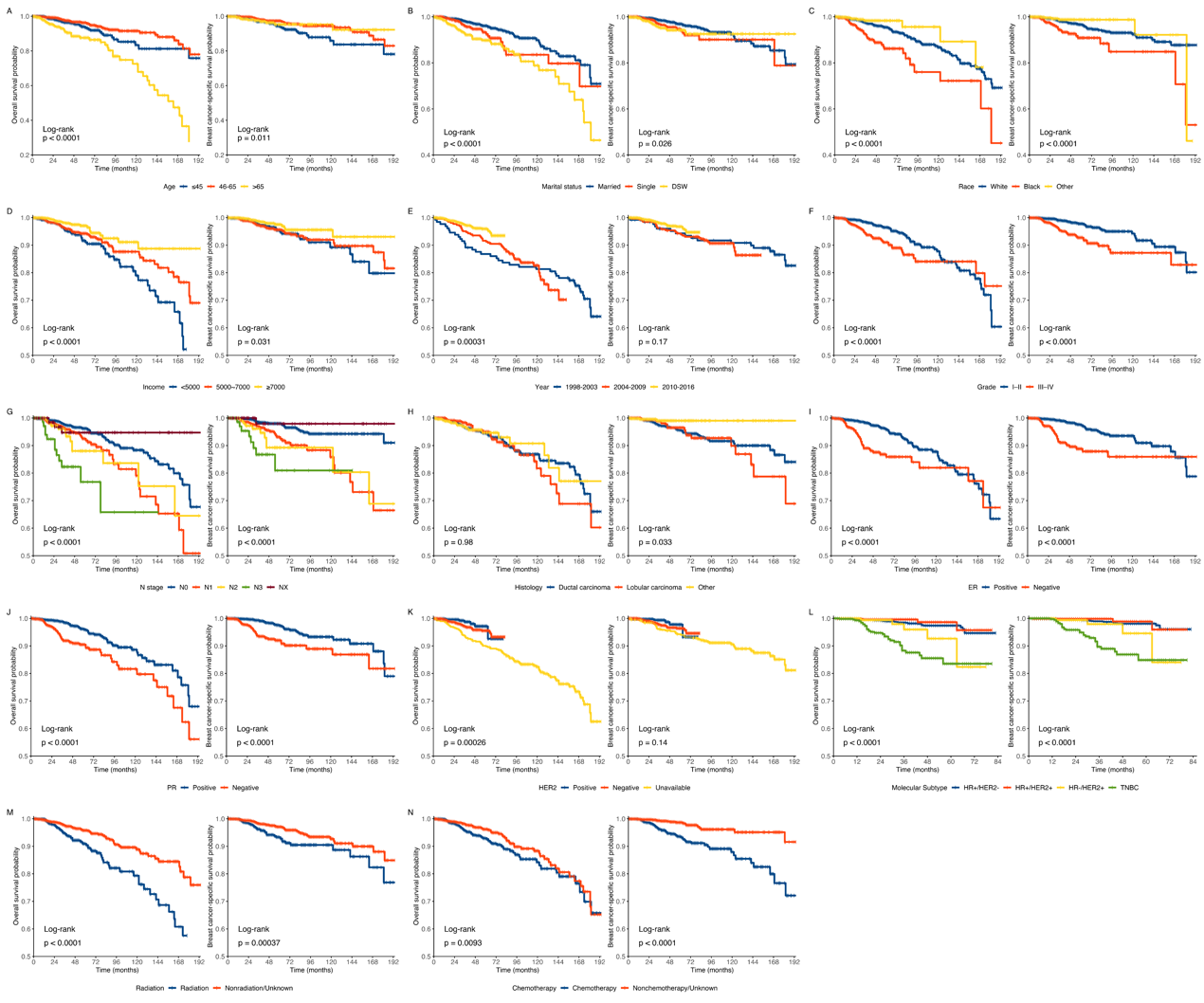


**Supplementary Figure 1**: The overall survival (OS) and breast cancer specific survival (BCSS) Kaplan-Meier curves of different demographic and clinicopathological characteristics of breast cancer patients treated with NSM. A) Age, B) Marital status, C) Race, D) Income, E) Year of diagnosis, F) Tumor grade, G) N stage, H) Histology, I) Estrogen Receptor (ER) status, J) Progesterone Receptor (PR) status, K) Human Epidermal Growth Receptor 2 (HER2) status, L) Molecular subtype, M) Radiation, N) Chemotherapy.


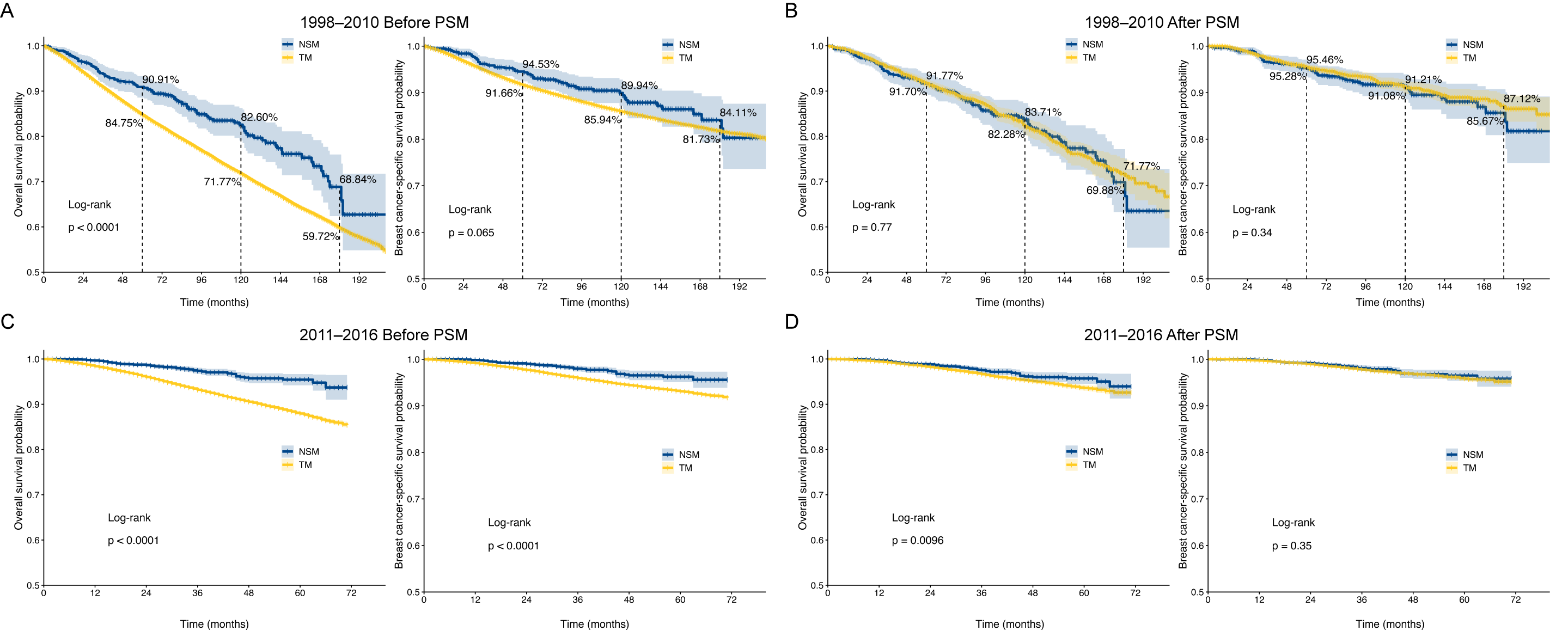


**Supplementary Figure 2:** Kaplan-Meier curves of overall survival (OS) and breast cancer-specific survival (BCSS) for breast cancer patients who underwent NSM and TM. **(A) (B)** OS/BCSS of patients in the original cohort and in the PSM cohort from 1998 to 2010. **(C) (D)** OS/BCSS of patients in the original cohort and in the PSM cohort from 2011 to 2016.
