# Supplementary material for: Prognosis Comparison Between Nipple-Sparing Mastectomy and Total Mastectomy in Breast Cancer: A Case-Control Study After Propensity Score Matching
Source: Ann Surg Oncol. 2021 Nov 20;29(4):2221–30. doi: 10.1245/s10434-021-11044-4 (PMC8933300; doi:10.1245/s10434-021-11044-4)
Supplement: Supplementary file 2 — Supplementary file2 (DOCX 125 KB) [file 10434_2021_11044_MOESM2_ESM.docx]

The detail information about this R package(MatchIt), please refer to the website.

(https://www.rdocumentation.org/packages/MatchIt/versions/4.2.0/topics/matchit)

**Setting the Treatment and Control Population (NSM and TM)**

**The factors in the propensity score model:**

Age, Marital status, Race, Median household incomes, Year of diagnosis, Grade, T stage, N stage, Histology, ER, PR, Molecular subtype.

**Regression model adopted:**

We used a **Logistic Regression model** to estimate the propensity scores.

**R code and fomula:**

matchit(Group ~ Age + Marriage + Race + Income + Year + Grade + T.stage + N.stage + ER + PR + Histology + Molecular.Subtype, data = rt, method ="nearest", ratio = 3, caliper = 0.02)

**The matching method to be used:**

The method is "nearest" for nearest neighbor matching (on the propensity score by default).

**The distance measure to be used:**

The default is "glm" for propensity scores estimated with logistic regression using "glm()".

**Ratio Setting:**

It means how many control units should be matched to each treated unit.

In our study, the ratio setting is Control(TM) 3 : Treated(NSM) 1.

**Regression model details:**

| Coefficients: | Estimate | Std.Error | t.value | Pr(>\|t\|) |  |
| --- | --- | --- | --- | --- | --- |
| (Intercept) | -4.51833 | 0.79205 | -5.705 | 1.17E-08 | *** |
| Age46-65 | -0.45321 | 0.0325 | -13.947 | <2e-16 | *** |
| Age>65 | -1.68334 | 0.05579 | -30.174 | <2e-16 | *** |
| Marriage.L | -0.22797 | 0.05507 | -4.139 | 3.48E-05 | *** |
| Marriage.Q | -0.01054 | 0.04832 | -0.218 | 0.827 |  |
| Marriage.C | -0.04058 | 0.04089 | -0.992 | 0.321 |  |
| RaceBlack | -0.0201 | 0.05328 | -0.377 | 0.706 |  |
| RaceOther | -0.07125 | 0.04514 | -1.578 | 0.114 |  |
| RaceUnknown | 0.11767 | 0.16672 | 0.706 | 0.48 |  |
| Income.L | 0.39887 | 0.03222 | 12.378 | <2e-16 | *** |
| Income.Q | -0.03752 | 0.02479 | -1.514 | 0.13 |  |
| Year2004-2009 | -0.17444 | 0.11634 | -1.499 | 0.134 |  |
| Year2010-2016 | 1.91372 | 0.11887 | 16.099 | <2e-16 | *** |
| GradeIII–IV | -0.05417 | 0.03688 | -1.469 | 0.142 |  |
| GradeUnknown | 0.09847 | 0.07305 | 1.348 | 0.178 |  |
| T.stageT1 | 0.50808 | 0.78047 | 0.651 | 0.515 |  |
| T.stageT2 | 0.35034 | 0.78048 | 0.449 | 0.654 |  |
| T.stageT3 | 0.1443 | 0.78185 | 0.185 | 0.854 |  |
| T.stageT4 | -0.34274 | 0.79315 | -0.432 | 0.666 |  |
| T.stageTX | 0.13345 | 0.85731 | 0.156 | 0.876 |  |
| N.stageN1 | 0.07838 | 0.03611 | 2.17 | 0.03 | * |
| N.stageN2 | 0.11425 | 0.07467 | 1.53 | 0.126 |  |
| N.stageN3 | -0.07055 | 0.11578 | -0.609 | 0.542 |  |
| N.stageNX | 0.40648 | 0.36174 | 1.124 | 0.261 |  |
| ERNegative | 0.01386 | 0.09545 | 0.145 | 0.885 |  |
| ERUnknown | 0.25493 | 0.23411 | 1.089 | 0.276 |  |
| PRNegative | -0.04733 | 0.04882 | -0.969 | 0.332 |  |
| PRUnknown | -0.25735 | 0.21512 | -1.196 | 0.232 |  |
| HistologyLobularcarcinoma | -0.02596 | 0.04185 | -0.62 | 0.535 |  |
| HistologyOther | -0.03179 | 0.0516 | -0.616 | 0.538 |  |
| Molecular.SubtypeLuminalB | -0.0361 | 0.04879 | -0.74 | 0.459 |  |
| Molecular.SubtypeHER2enriched | -0.07493 | 0.11828 | -0.633 | 0.526 |  |
| Molecular.SubtypeTNBC | -0.03766 | 0.10724 | -0.351 | 0.725 |  |
| Molecular.SubtypeUnknown | -0.33528 | 0.08312 | -4.034 | 5.49E-05 | *** |

Signif. codes: 0 ‘***’ 0.001 ‘**’ 0.01 ‘*’ 0.05 ‘.’ 0.1 ‘ ’ 1

(Dispersion parameter for binomial family taken to be 1)

Null deviance: 48093 on 140292 degrees of freedom

Residual deviance: 42342 on 140261 degrees of freedom

AIC: 42406

Number of Fisher Scoring iterations: 7

**Summary of Balance for All Data:**

|  | Means(Treated)Std. | Means(Control)Std. | Mean_Diff. | Var.Ratio | eCDFMean | eCDFMax |
| --- | --- | --- | --- | --- | --- | --- |
| distance | 0.081 | 0.0394 | 1.1263 | 0.8769 | 0.2705 | 0.4602 |
| Age≤45 | 0.3532 | 0.1984 | 0.3237 | . | 0.1547 | 0.1547 |
| Age46-65 | 0.5573 | 0.4925 | 0.1305 | . | 0.0648 | 0.0648 |
| Age>65 | 0.0895 | 0.309 | -0.769 | . | 0.2195 | 0.2195 |
| MarriageMarried | 0.669 | 0.5833 | 0.1823 | . | 0.0858 | 0.0858 |
| MarriageSingle | 0.1525 | 0.1376 | 0.0415 | . | 0.0149 | 0.0149 |
| MarriageDSW | 0.1436 | 0.2379 | -0.2687 | . | 0.0942 | 0.0942 |
| MarriageUnknown | 0.0349 | 0.0413 | -0.035 | . | 0.0064 | 0.0064 |
| RaceWhite | 0.7757 | 0.7969 | -0.0508 | . | 0.0212 | 0.0212 |
| RaceBlack | 0.0878 | 0.0917 | -0.014 | . | 0.004 | 0.004 |
| RaceOther | 0.1285 | 0.1059 | 0.0675 | . | 0.0226 | 0.0226 |
| RaceUnknown | 0.008 | 0.0054 | 0.0287 | . | 0.0026 | 0.0026 |
| Income<5000 | 0.157 | 0.2277 | -0.1944 | . | 0.0707 | 0.0707 |
| Income5000~7000 | 0.5023 | 0.4992 | 0.0063 | . | 0.0032 | 0.0032 |
| Income≥7000 | 0.3407 | 0.2731 | 0.1426 | . | 0.0676 | 0.0676 |
| Year1998-2003 | 0.0224 | 0.1291 | -0.7219 | . | 0.1068 | 0.1068 |
| Year2004-2009 | 0.0461 | 0.3028 | -1.2232 | . | 0.2566 | 0.2566 |
| Year2010-2016 | 0.9315 | 0.5681 | 1.4384 | . | 0.3634 | 0.3634 |
| GradeI–II | 0.6264 | 0.611 | 0.0318 | . | 0.0154 | 0.0154 |
| GradeIII–IV | 0.3263 | 0.3325 | -0.0134 | . | 0.0063 | 0.0063 |
| GradeUnknown | 0.0474 | 0.0565 | -0.043 | . | 0.0091 | 0.0091 |
| T.stageT0 | 0.0003 | 0.0004 | -0.0029 | . | 0.0001 | 0.0001 |
| T.stageT1 | 0.5807 | 0.544 | 0.0744 | . | 0.0367 | 0.0367 |
| T.stageT2 | 0.3297 | 0.3354 | -0.0121 | . | 0.0057 | 0.0057 |
| T.stageT3 | 0.0654 | 0.0718 | -0.0261 | . | 0.0064 | 0.0064 |
| T.stageT4 | 0.0095 | 0.0246 | -0.1552 | . | 0.0151 | 0.0151 |
| T.stageTX | 0.0142 | 0.0237 | -0.0798 | . | 0.0094 | 0.0094 |
| N.stageN0 | 0.6848 | 0.6923 | -0.016 | . | 0.0074 | 0.0074 |
| N.stageN1 | 0.2411 | 0.2175 | 0.0551 | . | 0.0236 | 0.0236 |
| N.stageN2 | 0.0432 | 0.0441 | -0.0047 | . | 0.0009 | 0.0009 |
| N.stageN3 | 0.0168 | 0.0224 | -0.0433 | . | 0.0056 | 0.0056 |
| N.stageNX | 0.0141 | 0.0237 | -0.0818 | . | 0.0096 | 0.0096 |
| ERPositive | 0.8125 | 0.7685 | 0.1126 | . | 0.044 | 0.044 |
| ERNegative | 0.1681 | 0.1803 | -0.0326 | . | 0.0122 | 0.0122 |
| ERUnknown | 0.0194 | 0.0512 | -0.2303 | . | 0.0318 | 0.0318 |
| PRPositive | 0.7207 | 0.6571 | 0.1418 | . | 0.0636 | 0.0636 |
| PRNegative | 0.2565 | 0.2812 | -0.0565 | . | 0.0247 | 0.0247 |
| PRUnknown | 0.0227 | 0.0617 | -0.2613 | . | 0.0389 | 0.0389 |
| HistologyDuctalcarcinoma | 0.7414 | 0.6997 | 0.0951 | . | 0.0416 | 0.0416 |
| HistologyLobularcarcinoma | 0.1663 | 0.1874 | -0.0567 | . | 0.0211 | 0.0211 |
| HistologyOther | 0.0923 | 0.1128 | -0.071 | . | 0.0205 | 0.0205 |
| Molecular.SubtypeLuminalA | 0.633 | 0.3781 | 0.5288 | . | 0.2549 | 0.2549 |
| Molecular.SubtypeLuminalB | 0.1155 | 0.0652 | 0.1575 | . | 0.0504 | 0.0504 |
| Molecular.SubtypeHER2enriched | 0.0453 | 0.0283 | 0.0816 | . | 0.017 | 0.017 |
| Molecular.SubtypeTNBC | 0.0945 | 0.0606 | 0.116 | . | 0.0339 | 0.0339 |
| Molecular.SubtypeUnknown | 0.1117 | 0.4678 | -1.1305 | . | 0.3561 | 0.3561 |

**Summary of Balance for Matched Data:**

|  | Means(Treated)Std | Means(Control)Std. | MeanDiff. | Var.Ratio | eCDFMean | eCDFMax | Std.Pair.Dist. |
| --- | --- | --- | --- | --- | --- | --- | --- |
| distance | 0.0809 | 0.0809 | 0 | 1.0001 | 0 | 0.001 | 0.0001 |
| Age≤45 | 0.3529 | 0.347 | 0.0125 | . | 0.006 | 0.006 | 0.0894 |
| Age46-65 | 0.5575 | 0.5653 | -0.0156 | . | 0.0078 | 0.0078 | 0.0869 |
| Age>65 | 0.0895 | 0.0877 | 0.0063 | . | 0.0018 | 0.0018 | 0.029 |
| MarriageMarried | 0.6689 | 0.6803 | -0.0242 | . | 0.0114 | 0.0114 | 0.1641 |
| MarriageSingle | 0.1525 | 0.1479 | 0.0129 | . | 0.0046 | 0.0046 | 0.1612 |
| MarriageDSW | 0.1437 | 0.1396 | 0.0115 | . | 0.004 | 0.004 | 0.1352 |
| MarriageUnknown | 0.0349 | 0.0322 | 0.0148 | . | 0.0027 | 0.0027 | 0.1321 |
| RaceWhite | 0.7758 | 0.7858 | -0.0239 | . | 0.0099 | 0.0099 | 0.1652 |
| RaceBlack | 0.0878 | 0.083 | 0.017 | . | 0.0048 | 0.0048 | 0.165 |
| RaceOther | 0.1284 | 0.1249 | 0.0105 | . | 0.0035 | 0.0035 | 0.1253 |
| RaceUnknown | 0.008 | 0.0064 | 0.0182 | . | 0.0016 | 0.0016 | 0.1099 |
| Income<5000 | 0.157 | 0.1572 | -0.0005 | . | 0.0002 | 0.0002 | 0.1067 |
| Income5000~7000 | 0.5025 | 0.5037 | -0.0024 | . | 0.0012 | 0.0012 | 0.1586 |
| Income≥7000 | 0.3404 | 0.3391 | 0.0029 | . | 0.0014 | 0.0014 | 0.1367 |
| Year1998-2003 | 0.0224 | 0.022 | 0.0027 | . | 0.0004 | 0.0004 | 0.0645 |
| Year2004-2009 | 0.0462 | 0.0488 | -0.0124 | . | 0.0026 | 0.0026 | 0.0427 |
| Year2010-2016 | 0.9315 | 0.9293 | 0.0087 | . | 0.0022 | 0.0022 | 0.0201 |
| GradeI–II | 0.6264 | 0.6351 | -0.0179 | . | 0.0087 | 0.0087 | 0.1545 |
| GradeIII–IV | 0.3264 | 0.3218 | 0.0097 | . | 0.0046 | 0.0046 | 0.1558 |
| GradeUnknown | 0.0472 | 0.0431 | 0.0193 | . | 0.0041 | 0.0041 | 0.1909 |
| T.stageT0 | 0.0003 | 0.0001 | 0.0124 | . | 0.0002 | 0.0002 | 0.0248 |
| T.stageT1 | 0.5806 | 0.5844 | -0.0076 | . | 0.0038 | 0.0038 | 0.1481 |
| T.stageT2 | 0.3299 | 0.3334 | -0.0075 | . | 0.0035 | 0.0035 | 0.1515 |
| T.stageT3 | 0.0654 | 0.0624 | 0.0122 | . | 0.003 | 0.003 | 0.1628 |
| T.stageT4 | 0.0095 | 0.0075 | 0.0214 | . | 0.0021 | 0.0021 | 0.1119 |
| T.stageTX | 0.0142 | 0.0123 | 0.0166 | . | 0.002 | 0.002 | 0.1358 |
| N.stageN0 | 0.6849 | 0.6952 | -0.0223 | . | 0.0104 | 0.0104 | 0.1756 |
| N.stageN1 | 0.2412 | 0.2381 | 0.0073 | . | 0.0031 | 0.0031 | 0.1601 |
| N.stageN2 | 0.0432 | 0.0398 | 0.0168 | . | 0.0034 | 0.0034 | 0.1411 |
| N.stageN3 | 0.0168 | 0.0144 | 0.0189 | . | 0.0024 | 0.0024 | 0.1376 |
| N.stageNX | 0.0139 | 0.0125 | 0.0118 | . | 0.0014 | 0.0014 | 0.1366 |
| ERPositive | 0.8126 | 0.8192 | -0.0169 | . | 0.0066 | 0.0066 | 0.1681 |
| ERNegative | 0.1681 | 0.1635 | 0.0125 | . | 0.0047 | 0.0047 | 0.1601 |
| ERUnknown | 0.0193 | 0.0174 | 0.0138 | . | 0.0019 | 0.0019 | 0.1194 |
| PRPositive | 0.7206 | 0.7282 | -0.0169 | . | 0.0076 | 0.0076 | 0.1577 |
| PRNegative | 0.2566 | 0.2516 | 0.0115 | . | 0.005 | 0.005 | 0.1636 |
| PRUnknown | 0.0227 | 0.0202 | 0.0171 | . | 0.0025 | 0.0025 | 0.1421 |
| HistologyDuctalcarcinoma | 0.7416 | 0.745 | -0.0077 | . | 0.0034 | 0.0034 | 0.1572 |
| HistologyLobularcarcinoma | 0.1662 | 0.168 | -0.0048 | . | 0.0018 | 0.0018 | 0.116 |
| HistologyOther | 0.0921 | 0.087 | 0.0178 | . | 0.0051 | 0.0051 | 0.1649 |
| Molecular.SubtypeLuminalA | 0.633 | 0.6435 | -0.0217 | . | 0.0105 | 0.0105 | 0.1283 |
| Molecular.SubtypeLuminalB | 0.1154 | 0.1087 | 0.021 | . | 0.0067 | 0.0067 | 0.1379 |
| Molecular.SubtypeHER2enriched | 0.0453 | 0.044 | 0.0064 | . | 0.0013 | 0.0013 | 0.0965 |
| Molecular.SubtypeTNBC | 0.0946 | 0.0932 | 0.0045 | . | 0.0013 | 0.0013 | 0.0927 |
| Molecular.SubtypeUnknown | 0.1117 | 0.1106 | 0.0035 | . | 0.0011 | 0.0011 | 0.0935 |

**Percent Balance Improvement:**

|  | Std.Mean.Diff. | Var.Ratio | eCDF.Mean | | eCDF.Max |
| --- | --- | --- | --- | --- | --- |
| distance | 100 | 99.9 | | 100 | 99.8 |
| Age≤45 | 96.1 | . | | 96.1 | 96.1 |
| Age46-65 | 88 | . | | 88 | 88 |
| Age>65 | 99.2 | . | | 99.2 | 99.2 |
| MarriageMarried | 86.7 | . | | 86.7 | 86.7 |
| MarriageSingle | 68.9 | . | | 68.9 | 68.9 |
| MarriageDSW | 95.7 | . | | 95.7 | 95.7 |
| MarriageUnknown | 57.7 | . | | 57.7 | 57.7 |
| RaceWhite | 53 | . | | 53 | 53 |
| RaceBlack | -21.3 | . | | -21.3 | -21.3 |
| RaceOther | 84.4 | . | | 84.4 | 84.4 |
| RaceUnknown | 36.6 | . | | 36.6 | 36.6 |
| Income<5000 | 99.8 | . | | 99.8 | 99.8 |
| Income5000~7000 | 61.5 | . | | 61.5 | 61.5 |
| Income≥7000 | 97.9 | . | | 97.9 | 97.9 |
| Year1998-2003 | 99.6 | . | | 99.6 | 99.6 |
| Year2004-2009 | 99 | . | | 99 | 99 |
| Year2010-2016 | 99.4 | . | | 99.4 | 99.4 |
| GradeI–II | 43.7 | . | | 43.7 | 43.7 |
| GradeIII–IV | 27.1 | . | | 27.1 | 27.1 |
| GradeUnknown | 55.1 | . | | 55.1 | 55.1 |
| T.stageT0 | -324.7 | . | | -324.7 | -324.7 |
| T.stageT1 | 89.8 | . | | 89.8 | 89.8 |
| T.stageT2 | 38 | . | | 38 | 38 |
| T.stageT3 | 53.3 | . | | 53.3 | 53.3 |
| T.stageT4 | 86.2 | . | | 86.2 | 86.2 |
| T.stageTX | 79.2 | . | | 79.2 | 79.2 |
| N.stageN0 | -39.2 | . | | -39.2 | -39.2 |
| N.stageN1 | 86.8 | . | | 86.8 | 86.8 |
| N.stageN2 | -260 | . | | -260 | -260 |
| N.stageN3 | 56.4 | . | | 56.4 | 56.4 |
| N.stageNX | 85.6 | . | | 85.6 | 85.6 |
| ERPositive | 85 | . | | 85 | 85 |
| ERNegative | 61.5 | . | | 61.5 | 61.5 |
| ERUnknown | 94 | . | | 94 | 94 |
| PRPositive | 88.1 | . | | 88.1 | 88.1 |
| PRNegative | 79.6 | . | | 79.6 | 79.6 |
| PRUnknown | 93.5 | . | | 93.5 | 93.5 |
| HistologyDuctalcarcinoma | 91.9 | . | | 91.9 | 91.9 |
| HistologyLobularcarcinoma | 91.5 | . | | 91.5 | 91.5 |
| HistologyOther | 74.9 | . | | 74.9 | 74.9 |
| Molecular.SubtypeLuminalA | 95.9 | . | | 95.9 | 95.9 |
| Molecular.SubtypeLuminalB | 86.7 | . | | 86.7 | 86.7 |
| Molecular.SubtypeHER2enriched | 92.2 | . | | 92.2 | 92.2 |
| Molecular.SubtypeTNBC | 96.1 | . | | 96.1 | 96.1 |
| Molecular.SubtypeUnknown | 99.7 | . | | 99.7 | 99.7 |


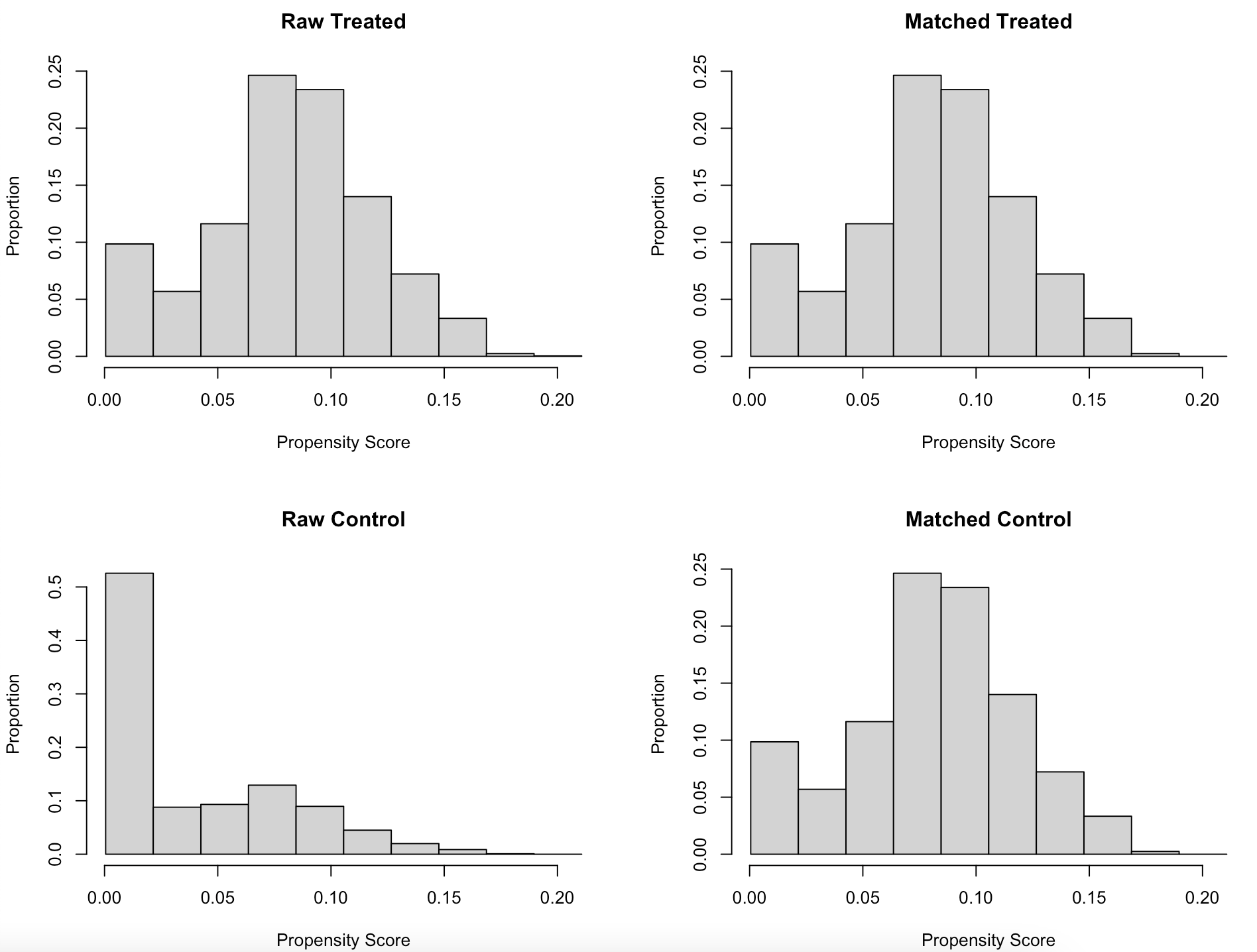


**Sample sizes summary:**

|  | Control (TM) | Treated (NSM) |
| --- | --- | --- |
| All | 134528 | 5765 |
| Matched | 17289 | 5763 |
| Unmatched | 117239 | 2 |
| Discarded | 0 | 0 |
